# Supplementary material for: Split-Small GTPase Reassembly as a Method to Control Cellular Signaling with User-Defined Inputs
Source: ACS Chem Biol. 2025 Aug 26;20(9):2049–55. doi: 10.1021/acschembio.5c00083 (PMC12442047; doi:10.1021/acschembio.5c00083)
Supplement: Supplementary file 1 [file cb5c00083_si_001.pdf]

Supporting Information

Split-Small GTPase Reassembly as a Method to Control Cellular Signaling with User-Defined Inputs

Yuchen He,[1] Benjamin M. Faulkner,[1] Rachel S. Weatherford,[1] Emily Hyun,[1] and Cliff I. Stains[1,2,3,*]

[1]Department of Chemistry, University of Virginia, Charlottesville, VA 22904, USA

[2]University of Virginia Cancer Center, University of Virginia, Charlottesville, VA 22908, USA

[3]Virginia Drug Discovery Consortium, Blacksburg, VA 24061, USA

*Corresponding author, E-mail: cstains@virginia.edu

Table of Contents:

Figure S1 2
Figure S2 3
Figure S3 4
Figure S4 5
Figure S5 6
Figure S6 7
Figure S7 8
Materials and Methods 9
Table S1 16
Table S2 17
References 25

Figure S1

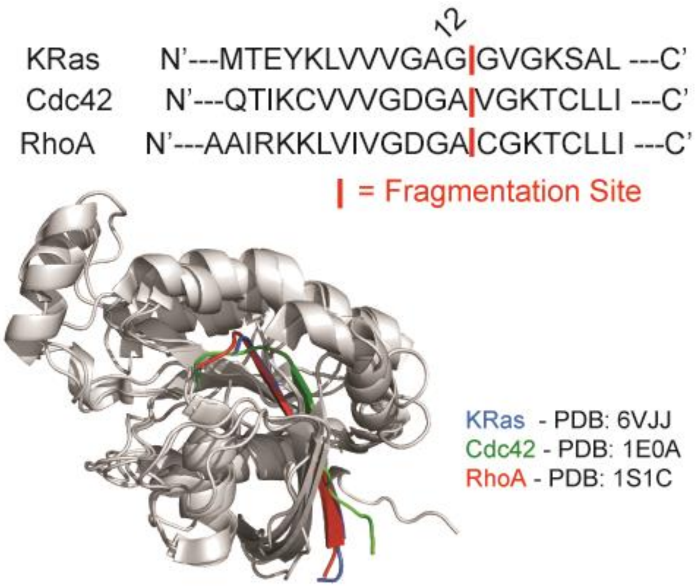

Figure S1. Application of the N12/13C fragmentation site across the small GTPase superfamily. A sequence alignment for KRas, Cdc42, and RhoA showing the N12/13C fragmentation site is shown (top). Numbering refers to Cdc42. An overlay of crystal structures corresponding to the indicated, active small GTPase is shown (bottom). The N12 fragment of each small GTPase is highlighted.

Figure S2

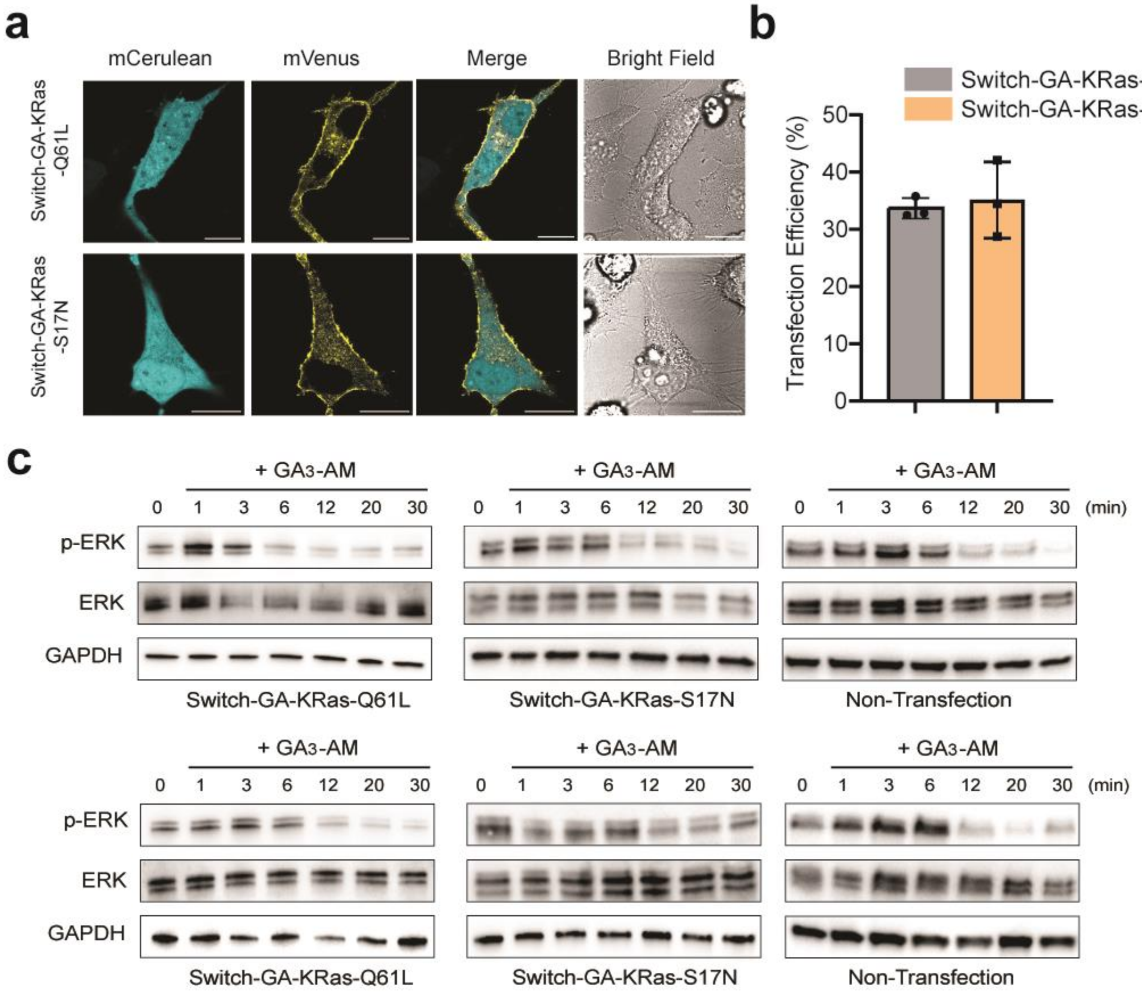

Figure S2. Switch-GA-Split-KRas activates ERK signaling. **a)** Confocal images of HeLa cells expressing Switch-GA-KRas-Q61L or Switch-GA-KRas-S17N constructs. mCerulean fluorescence is observed in the cytosol while the mVenus fluorescence is localized to the cell membrane. Scale bar represents 20 μm. **b)** Transfection efficiency of Switch-GA-Split-KRas constructs in HeLa cells was determined by normalizing the number of fluorescent cells to the total observed cells in the bright field channel. Data points are biological replicates in which > 50 cells are imaged in each replicate. **c)** Western blots for biological replicates assessing phosphorylation of ERK using Switch-GA-Split-KRas. HeLa cells were stimulated with 10 μM gibberellin (GA_3-AM) for the indicated time, lysed, and Western blots were performed with the indicated antibody.

Figure S3

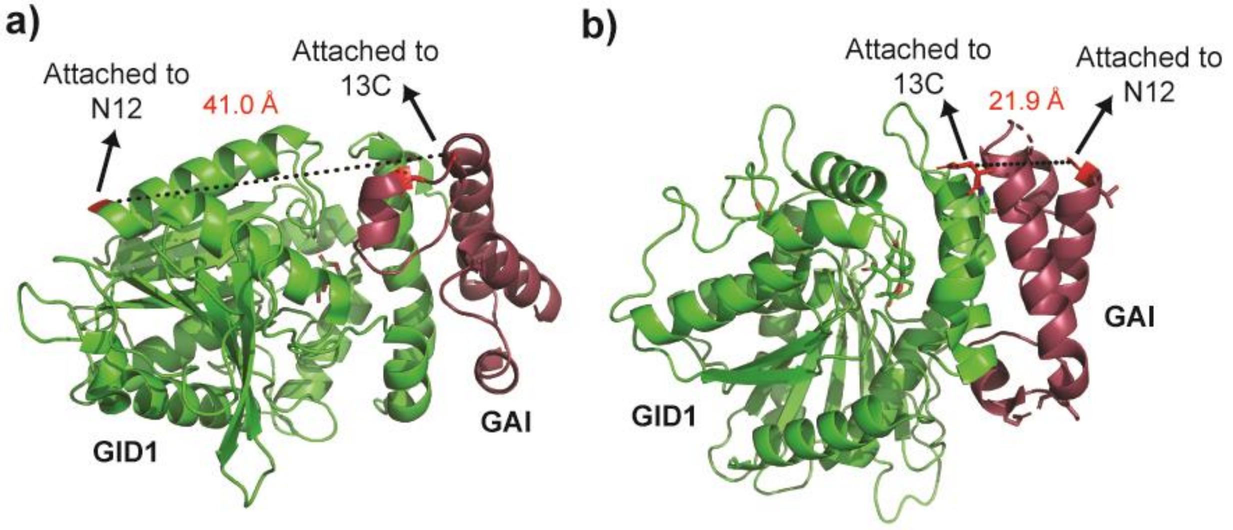

Figure S3. The termini of the GAI and GID1 are on the same face of the GA-mediated complex (PDB: 2ZSH). **a)** GAI and GID1 termini used for fusion in the GA-KRas-Q61L construct. **b)** GAI and GID1 termini used for fusion in the Switch-GA-KRas-Q61L construct. Fusion of these termini to either N12 or 13C using flexible 17-30 amino acid linkers does not appreciably influence reassembly (see **Figure 2**).

Figure S4

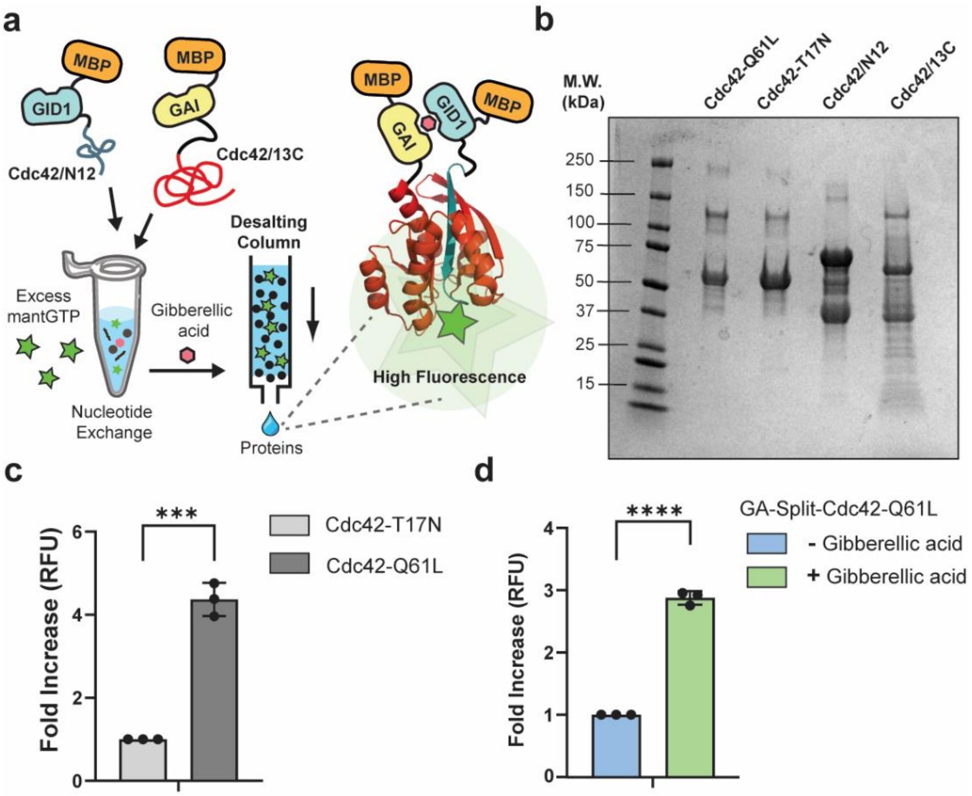

Figure S4. An *in vitro* reassembly assay directly demonstrates GA-gated activation of split-Cdc42. **a)** Schematic of the *in vitro* GA-gated split-Cdc42 reassembly assay. Equal concentrations of GA-Split-Cdc42 fragments are incubated with gibberellic acid (GA), followed by addition of a 10-fold excess of mantGTP to initiate nucleotide exchange. The reaction is quenched with excess Mg^{2+} to lock mantGTP in the GTPase binding pocket, and unbound mantGTP is removed by desalting. Reassembled split-Cdc42 binds mantGTP, leading to an increase in fluorescence. **b)** SDS-PAGE analysis of purified recombinant proteins used in this assay. Full-length controls: MBP-Cdc42-Q61L (63.2 kDa) and MBP-Cdc42-T17N (63.3 kDa). Split-Cdc42 constructs: MBP-GID1-Cdc42/N12 (84.2 kDa) and Cdc42/13C-GAI-MBP (74.4 kDa). **c)** mantGTP binding assay with full-length Cdc42 proteins (5 µM) incubated with 50 µM mantGTP. A 4.4-fold fluorescence increase is observed in constitutively active Cdc42-Q61L compared to the dominant negative mutant Cdc42-T17N, validating the assay's ability to detect differential nucleotide binding. **d)** GA-dependent reassembly of GA-Split-Cdc42-Q61L assessed by mantGTP binding. Split fragments (20 µM each) were incubated with or without 50 µM GA in the presence of 200 µM mantGTP. A 2.9-fold fluorescence increase was observed upon GA addition, indicating reassembly of split-Cdc42. Data represent means ± SD from three technical replicates. Statistical differences were determined using a two-tailed, unpaired Student's *t*-test. *** indicates a p-value of <0.001 and **** indicates a p-value of <0.0001.

Figure S5

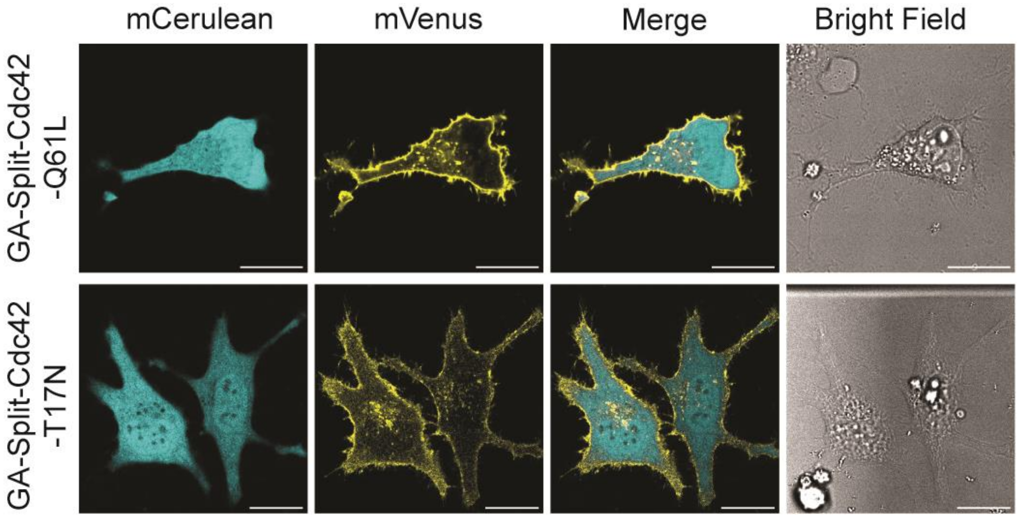

Figure S5. Confocal images of HeLa cells expressing the GA-Split-Cdc42 fragments. mCerulean fluorescence is observed in the cytosol while the mVenus fluorescence is localized to the cell membrane for both GA-Split-Cdc42-Q61L and GA-Split-Cdc42-T17N.

Figure S6

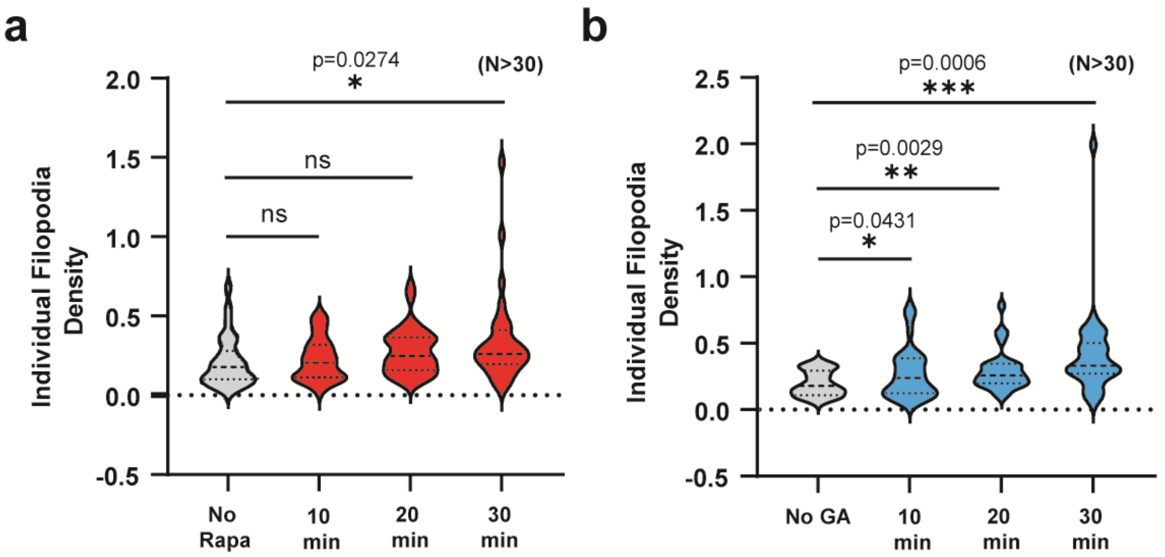

Figure S6. Time-course comparison of rapamycin-gated and gibberellic acid-gated filopodia formation using split-Cdc42-Q61L. **a)** Filopodia density in HeLa cells transfected with Rapa-Split-Cdc42-Q61L was measured at the indicated time points after rapamycin stimulation. **b)** Filopodia density in HeLa cells transfected with GA-Split-Cdc42-Q61L was assessed following stimulation with GA_3-AM for the indicated time. Filopodia density was calculated as the ratio of filopodia number divided by the total cell edge length, using FiloQuant.[1] Data from over 30 transfected cells per group were combined to generate the violin plots above. Statistical differences were determined using a two-tailed, unpaired Student's *t*-test. ns indicates a p-value of >0.05, * indicates a p-value of <0.05, ** indicates a p-value of <0.01, and *** indicates a p-value of <0.001. On average, a more rapid turn-on is observed in the GA-gated system.

Figure S7

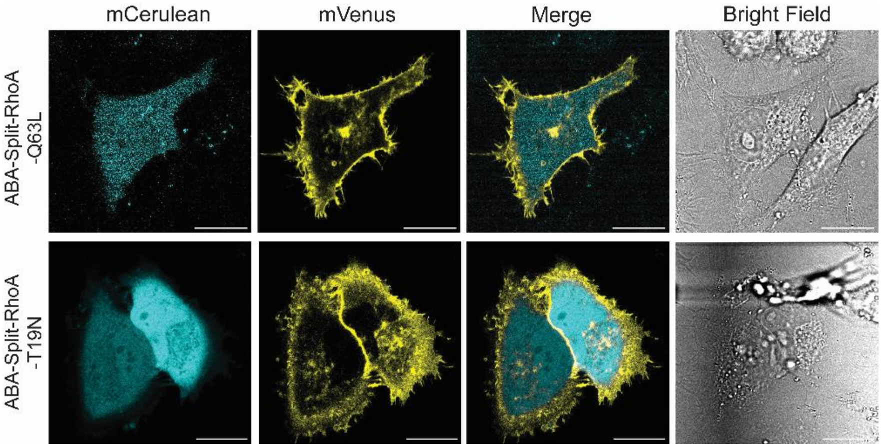

Figure S7. Localization of ABA-gated split-RhoA constructs in HeLa cells. Confocal images of HeLa cells expressing ABA-Split-RhoA-Q63L or ABA-Split-RhoA-T19N constructs. mCerulean fluorescence is localized to the cytosol while mVenus fluorescence is predominantly observed at the plasma membrane. Scale bar represents 20 μm.

Materials and Methods

Instrumentation and reagents

Polymerase chain reaction (PCR) was performed using an Eppendorf thermocycler (05-414-456). Western blots were imaged with a Bio-Rad ChemiDoc XRS+ gel imager (1708265). Protein concentrations were measured using a Synergy H1 hybrid plate reader from Thermo Fisher (11-120-533) for Bradford assays. Mammalian cells were counted with an Invitrogen Countess™ 3 automated cell counter (AMQAX2000). Confocal microscopy was conducted on a Leica STELLARIS 8 system, which features confocal/FLIM/tauSTED capabilities and a tunable white light laser. Cell culture medium was DMEM (Dulbecco's Modified Eagle Medium, Thermo Fisher, 10569010), supplemented with 10% (v/v) fetal bovine serum, 100 U/ml penicillin and 100 g/ml streptomycin. Additional reagents included Gibco™ DPBS (Thermo Fisher, 14-040-133), Gibco™ DMEM (Thermo Fisher, 21-063-029), Gibco™ Opti-MEM™ I Reduced Serum Medium (Thermo Fisher, 11-058-021), and DMSO (Sigma-Aldrich, D8418-100ML). Molecular biology enzymes and kits employed were Platinum Taq DNA polymerase High Fidelity (Thermo Fisher, 11304011), restriction enzymes from New England Biolabs (NEB), T4 DNA ligase (NEB, M0202M), Gibson Assembly Master Mix (NEB, E2611S), and Qiagen plasmid preparation kits (Miniprep Kit, 27104; Maxi Kit, 12162). *In vitro* reassembly assays and protein purification used mantGTP (2'-(or-3')-O-(N-Methylanthraniloyl) Guanosine 5'-Triphosphate, Trisodium Salt) (Thermo Fisher, M12415), Zeba™ desalt spin column (Thermo Fisher, 89892), Isopropyl-β-D-thiogalactopyranoside (IPTG) (Chem Impex, 00194), B-PER™ complete bacterial protein extraction reagent (Thermo Fisher, 89821), Econo-Pac Columns (Bio-Rad, 7321010), Amylose Resin (NEB, E8021S), Amicon Ultra-15 Centrifugal Filter Unit, Ultracel, 3 KDa (EMD Millipore, UFC900324), Snakeskin™ Dialysis Tubing, 10K MWCO (Thermo Fisher, 68100). Chemical inducers such as abscisic acid (TCI Chemicals, A1698-100MG) and gibberellic acid acetoxymethyl ester (GA_3-AM, Millipore Sigma, SML1959-50MG) were utilized. For *in vitro*

mantGTP binding assays, the acid form of gibberellic acid (Millipore Sigma, 48880) was used. *In vitro* mantGTP assays were conducted in 96-well half-area black flat bottom polystyrene plates (Sigma-Aldrich, CLS3694-100EA). For cell lysis, protease inhibitor cocktail III (10 µl/ml, Calbiochem, 539134) and phosphatase inhibitor cocktail 1 (10 µl/ml, Sigma, P2825) were used in the lysis buffer. The Bradford protein assay kit from Bio-Rad (5000201) was used for determinations of total protein concentration in lysates. Western blotting employed antibodies from Cell Signaling Technology for Phospho-p44/42 MAPK (Erk1/2) (4377S), P44/42 MAPK (Erk1/2) (9102S), GAPDH (3683S) and HRP-conjugated goat anti-rabbit IgG (7074S). Blots were visualized using Thermo Fisher's SuperSignal® West Dura Extended Duration Chemiluminescent Substrate (34076).

Cloning

Constructs used for mammalian transfection were primarily prepared using multiple overlap extension PCR steps followed by restriction enzyme-based cloning. In cases where overlap extension PCR was challenging due to repetitive linker sequences, Gibson assembly was utilized.[2] The mCerulean and mVenus sequences were sourced from the mCerulean N1 (Addgene, 27795) and mVenus C1 (Addgene, 27794) plasmids, gifts from Steven Vogel at the National Institutes of Health. The N12 and 13C fragments of each small GTPase originated from previously published vectors.[3] GID1 and GAI_{1-92} were amplified from the pSLQ2812 pPB: CAG-GID1-VPR-IRES-Puro-WPRE PGK-GAI-tagBFP-SpdCas9 plasmid (Addgene, 84240), while PYL and ABI were amplified from the Pslq2817 pPB: CAG-PYL1-VPR-IRES-Puro-WPRE-SV40PA PGK-ABI-tagBFP-SpdCas9 plasmid (Addgene, 84239), both generously provided by Stanley Qi at Stanford University. Plasmids designed for recombinant protein expression were built using pMAL-c5X-His (Genophore, GV03006) or pET21b vector containing a C-terminal MBP purification tag. All plasmids constructed were verified through DNA sequencing. Plasmid information and protein sequences are given in **Tables S1** and **S2**.

Mammalian cell culture and plasmid transfection

HeLa cells (ATCC, CCL-2) were cultured in Dulbecco's modified Eagle's medium (DMEM) supplemented with 10% (v/v) fetal bovine serum containing 100 U/ml penicillin and 100 g/ml streptomycin. Cells were maintained at 37°C in a 5% CO_2 humidified incubator. For plasmid transfection, cells were seeded at 1.2 x 10^5 cells/cm^2 either on tissue culture treated dishes (Thomas Scientific, 1228K66) for western blot analysis, or on 35 mm poly-d-lysine coated glass-bottom dishes (MatTek Corporation, NC9005934) for confocal microscopy. After 24 hours, the cells had reached ~70-90% confluency and transient transfection was performed following the manufacturer's instructions (Invitrogen, L3000015).

Confocal fluorescence imaging

Confocal images were captured using a Leica STELLARIS 8 confocal/FLIM/tauSTED microscope system equipped with tunable white light lasers and a 37-2 digital temperature controller. The LAS-AF software facilitated image acquisition, with subsequent processing in Fiji (ImageJ). Cells were pre-stained with Hoechst 33342 (Thermo Fisher, H3570) and CellMaskTM Deep Red Actin Tracking Stain (Thermo Fisher, A57245), following the manufacturer's guidelines, to visualize nuclei and cytoskeletal structures. After staining, cells were rinsed three times with pre-warmed DPBS and incubated in 1.5 mL of DMEM containing 25 mM HEPES (Thermo Fisher, 21-063-029) for imaging. For image acquisition post-CID stimulation, 0.5 ml of DMEM with 4X concentrations of ABA or GA_3-AM was added dropwise to the dishes using a syringe. Final working concentrations were 100 µM ABA and 10 µM GA_3-AM, consistent with prior studies that successfully used these levels to activate the PYL-ABI and GID1-GAI systems in mammalian cells.[4, 5] These concentrations were selected based on their demonstrated ability to induce robust signaling responses while maintaining cell viability. Fluorophores were excited with a diode laser at 405 nm or a white light laser spanning 440-790 nm. Nucleus staining was detected with a PMT detector set to capture emission at 430-460 nm following excitation at 405

nm. Actin was visualized with excitation at 652 nm and emission detected between 660-710 nm. mCerulean fluorescence was captured with 458 nm excitation and 468-508 nm emission, while mVenus fluorescence was observed using 514 nm excitation with emission detected at 520-560 nm.

Lysate preparation and western blot

Transiently transfected HeLa cells were serum starved overnight in Opti-MEM before being stimulated with GA_3-AM. Cells were exposed to 10 μM GA_3-AM in pre-warmed Opti-MEM for the indicated time, followed by rinsing with ice-cold DPBS. Subsequently, cells were lysed using the following lysis buffer: 50 mM Tris-Cl (pH = 7.5 at 25°C), 150 mM NaCl, 50 mM β-glycerophosphate, 10 mM sodium pyrophosphate, 30 mM NaF, 1% Triton X-100, 2 mM EGTA, 100 μM Na_3VO_4, 1 mM DTT, protease inhibitor cocktail III (10 μl/ml, Calbiochem, 539134), and phosphatase inhibitor cocktail 1 (10 μl/ml, Sigma, P2825). The lysates were chilled on ice for 15 minutes before centrifugation at 17,000 g for 10 min at 4°C. Protein concentrations were quantified using a Bradford assay (Bio-Rad, 5000201) and normalized for subsequent experiments. For western blot analysis, 5-20 μg of total protein from each lysate was resolved on 12% SDS-PAGE gels and transferred to nitrocellulose membranes. The membranes were then blocked and incubated with the indicated primary antibodies, followed by detection using enhanced chemiluminescence (Thermo Scientific, 34076).

Recombinant protein expression and purification

Plasmids encoding MBP-tagged Cdc42 constructs were transformed into the *Escherichia coli* BL21-Gold (DE3) strain. A single colony was used to inoculate 1 L of terrific broth (TB), and cultures were grown at 37°C with shaking until mid-log phase (OD_{600} ~0.6). Protein expression was induced with 0.5 mM IPTG, followed by continued incubation at 37°C for 3 hr. Cultures were then shifted to 18°C for overnight expression (~18 hr). Cells were harvested by

centrifugation at 3220 g for 35 min at 4°C. The resulting cell pellets were lysed using the B-PER™ complete bacterial protein extraction reagent (Thermo Fisher, 89821) and clarified by centrifugation at 15,000 g for 20 min at 4°C. The supernatant was then diluted with six volumes of 1X column buffer (20 mM Tris-Cl, pH 7.4 at 25°C; 200 mM NaCl; 1 mM EDTA) and purified using amylose resin (NEB, E8021S) using gravity flow (0.5 mL resin per 1L culture). Columns were washed with 100 mL column buffer, and bound proteins were eluted with 10 mM maltose in 10 mL of column buffer. Eluates were concentrated using ultra-15 centrifugal filter unit (EMD Millipore, UFC900324) and dialyzed into GTPase storage buffer (50 mM Tris-Cl, pH 7.5 at 25°C; 150 mM NaCl; 20% glycerol).

Cdc42/13C-GAI-MBP protein was predominantly expressed in inclusion bodies. Pellets containing inclusion bodies were solubilized in column buffer supplemented with 8 M urea and were then loaded into Snakeskin dialysis tubing and refolded using stepwise dialysis, gradually reducing the urea concentration (4 M, 2 M, 1 M, 0.5 M). Refolded proteins were purified using amylose resin, following the same elution and concentration steps as above, and dialyzed into GTPase storage buffer. Final protein concentrations were measured using a NanoDrop spectrophotometer.

***In vitro* assay for GA-gated split-Cdc42 reassembly**

To evaluate chemically induced reassembly of split-Cdc42, purified protein fragments were incubated with a fluorescent GTP analog, mantGTP (Thermo Fisher, M12415), in nucleotide exchange buffer (20 mM Tris-Cl, pH 7.5 at 25°C; 50 mM NaCl, 5 mM EDTA, 1% glycerol). For full-length Cdc42 controls, both constitutively active and dominant negative mutants were used at a final concentration of 5 µM, with 50 µM mantGTP. In the split-Cdc42 condition, 20 µM of each protein fragment (N12 and 13C) was combined in the absence or presence of GA (50 µM) and incubated with 200 µM mantGTP. The nucleotide exchange reaction was conducted at

room temperature for 30 min with gentle shaking. The nucleotide exchange reaction was quenched by adding excess $MgCl_2$ (1.33 M) and placing the reaction on ice. Unbound nucleotide was removed using Zeba™ spin desalting columns (Thermo Fisher, 89892), pre-equilibrated with wash buffer (20 mM Tris-Cl, pH 7.5 at 25°C; 50 mM NaCl; 10 mM $MgCl_2$; 1% glycerol). Eluted samples were transferred to a black 96-well plate, and fluorescence was measured using a plate reader (λex = 355 nm, λem = 448 nm) over the course of 1 hr at 30°C. For analysis, fluorescence values at 10 minutes after incubation at 30°C were used, with background subtraction using the mantGTP-only controls. Data were normalized to dominant negative controls within each experimental group and standard deviations were calculated using error propagation.

Data analysis

To detect and quantify Cdc42-induced filopodia formation, we utilized the open-source ImageJ plugin, FiloQuant.[1] This tool enables the extraction of quantitative data, such as the number and length of filopodia and cell edge length.[6] We evaluated the number of filopodia per cell to assess the efficacy of our GA-Split-Cdc42 system on filopodia formation. Data from over 50 transfected cells, collected across multiple cell culture dishes, were aggregated and analyzed. For fold-change comparisons, the mean values of filopodia number or cell retraction percentage before and after treatment were used to calculate fold increases. To estimate the standard deviation (SD) of the fold-change, we applied error propagation, combining the SDs of the pre- and post-treatment groups using standard propagation of uncertainty formulas. For the time course comparison between GA-Split-Cdc42 and Rapa-Split-Cdc42 systems, we collected cell images at different time points after CID stimulation (10, 20, and 30 min). Filopodia density, defined as the ratio of filopodia number to total cell edge length, was used to gauge the efficiency of split-Cdc42 systems. For the assessment of ABA-Split-RhoA signaling, the cell area was measured using ImageJ to define the region of interest (ROI) for the same transfected cell

before and after ABA stimulation. Data for each ABA-Split-RhoA construct was gathered from more than 50 cells from multiple cell culture dishes. Statistical analyses were conducted using unpaired, two-tailed Student's *t*-tests (GraphPad Prism 9.5, GraphPad Software).

Table S1. Construct description and Addgene IDs.

Construct	Description	Addgene ID
Switch-GA-KRas-Q61L	pIRES-mCerulean-GAI1-92-KRas/N12-KRas/13C(Q61L)-GID1-mVenus-CAAX	232262
Switch-GA-KRas-S17N	pIRES-mCerulean-GAI1-92-KRas/N12-KRas/13C(S17N)-GID1-mVenus-CAAX	232263
GA-Split-Cdc42-Q61L	pIRES-mCerulean-GID1-Cdc42/N12-Cdc42/13C(Q61L)-GAI1-92-mVenus-CAAX	232264
GA-Split-Cdc42-T17N	pIRES-mCerulean-GID1-Cdc42/N12-Cdc42/13C(T17N)-GAI1-92-mVenus-CAAX	232265
ABA-Split-RhoA-Q63L	pIRES-mCerulean-ABI-RhoA/N12-RhoA/13C(Q63L)-PYL-mVenus-CAAX	232266
ABA-Split-RhoA-T19N	pIRES-mCerulean-ABI-RhoA/N12-RhoA/13C(T19N)-PYL-mVenus-CAAX	232267

Table S2. Amino Acid Sequences for Constructs Used in This Work

1) **pIRES-mCerulean-GAI$_{1-92}$-KRas/N12-KRas/13C(Q61L)-GID1-mVenus-CAAX**

> 61L is underlined.

MVSKGEELFTGVVPILVELDGDVNGHKFSVSGEGEGDATYGKLTLKFICTTGKLPVPWPTLVTT
LTWGVQCFARYPDHMKQHDFFKSAMPEGYVQERTIFFKDDGNYKTRAEVKFEGDTLVNRIELK
GIDFKEDGNILGHKLEYNAISDNVYITADKQKNGIKANFKIRHNIEDGSVQLADHYQQNTPIGDGP
VLLPDNHYLSTQSKLSKDPNEKRDHMVLLEFVTAAGITLGMDELYKGGGSSGGGMKRDHHHH
HHQDKKTMMMNEEDDGNGMDELLAVLGYKVRSSEMADVAQKLEQLEVMMSNVQEDDLSQLA
TETVHYNPAELYTWLDSMLTDLNQISYASRGGGSSGGGELMTEYKLVVVGAG∗---***IRES Region***---
MGVGKSALTIQLIQNHFVDEYDPTIEDSYRKQVVIDGETCLLDILDTAG**L**EEYSAMRDQYMRTGE
GFLCVFAINNTKSFEDIHHYREQIKRVKDSEDVPMVLVGNKCDLPSRTVDTKQAQDLARSYGIP
FIETSAKTRQGVDDAFYTLVREIRKHKEKMSKDGGDPNWELVYTARLQGGGSSGGGQISYASR
GMAASDEVNLIESRTVVPLNTWVLISNFKVAYNILRRPDGTFNRHLAEYLDRKVTANANPVDGV
FSFDVLIDRRINLLSRVYRPAYADQEQPPSILDLEKPVDGDIVPVILFFHGGSFAHSSANSAIYDTL
CRRLVGLCKCVVVSVNYRRAPENPYPCAYDDGWIALNWVNSRSWLKSKKDSKVHIFLAGDSS
GGNIAHNVALRAGESGIDVLGNILLNPMFGGNERTESEKSLDGKYFVTVRDRDWYWKAFLPEG
EDREHPACNPFSPRGKSLEGVSFPKSLVVVAGLDLIRDWQLAYAEGLKKAGQEVKLMHLEKAT
VGFYLLPNNNHFHNVMDEISAFVNAECGGGSSGGGVSKGEELFTGVVPILVELDGDVNGHKFS
VSGEGEGDATYGKLTLKLICTTGKLPVPWPTLVTTLGYGLQCFARYPDHMKQHDFFKSAMPEG
YVQERTIFFKDDGNYKTRAEVKFEGDTLVNRIELKGIDFKEDGNILGHKLEYNYNSHNVYITADK
QKNGIKANFKIRHNIEDGGVQLADHYQQNTPIGDGPVLLPDNHYLSYQSKLSKDPNEKRDHMV
LLEFVTAAGITLGMDELYKKKKKKKSKTKCVIM∗

2) **pIRES-mCerulean-GAI$_{1-92}$-KRas/N12-KRas/13C(S17N)-GID1-mVenus-CAAX**

> 17N is underlined.

MVSKGEELFTGVVPILVELDGDVNGHKFSVSGEGEGDATYGKLTLKFICTTGKLPVPWPTLVTT
LTWGVQCFARYPDHMKQHDFFKSAMPEGYVQERTIFFKDDGNYKTRAEVKFEGDTLVNRIELK
GIDFKEDGNILGHKLEYNAISDNVYITADKQKNGIKANFKIRHNIEDGSVQLADHYQQNTPIGDGP
VLLPDNHYLSTQSKLSKDPNEKRDHMVLLEFVTAAGITLGMDELYKGGGSSGGGMKRDHHHH
HHQDKKTMMMNEEDDGNGMDELLAVLGYKVRSSEMADVAQKLEQLEVMMSNVQEDDLSQLA
TETVHYNPAELYTWLDSMLTDLNQISYASRGGGSSGGGELMTEYKLVVVGAG*---***IRES Region***---
MGVGK**N**ALTIQLIQNHFVDEYDPTIEDSYRKQVVIDGETCLLDILDTAGQEEYSAMRDQYMRTG
EGFLCVFAINNTKSFEDIHHYREQIKRVKDSEDVPMVLVGNKCDLPSRTVDTKQAQDLARSYGI
PFIETSAKTRQGVDDAFYTLVREIRKHKEKMSKDGGDPNWELVYTARLQGGGSSGGGQISYAS
RGMAASDEVNLIESRTVVPLNTWVLISNFKVAYNILRRPDGTFNRHLAEYLDRKVTANANPVDG
VFSFDVLIDRRINLLSRVYRPAYADQEQPPSILDLEKPVDGDIVPVILFFHGGSFAHSSANSAIYDT
LCRRLVGLCKCVVVSVNYRRAPENPYPCAYDDGWIALNWVNSRSWLKSKKDSKVHIFLAGDSS
GGNIAHNVALRAGESGIDVLGNILLNPMFGGNERTESEKSLDGKYFVTVRDRDWYWKAFLPEG
EDREHPACNPFSPRGKSLEGVSFPKSLVVVAGLDLIRDWQLAYAEGLKKAGQEVKLMHLEKAT
VGFYLLPNNNHFHNVMDEISAFVNAECGGGSSGGGVSKGEELFTGVVPILVELDGDVNGHKFS
VSGEGEGDATYGKLTLKLICTTGKLPVPWPTLVTTLGYGLQCFARYPDHMKQHDFFKSAMPEG
YVQERTIFFKDDGNYKTRAEVKFEGDTLVNRIELKGIDFKEDGNILGHKLEYNYNSHNVYITADK
QKNGIKANFKIRHNIEDGGVQLADHYQQNTPIGDGPVLLPDNHYLSYQSKLSKDPNEKRDHMV
LLEFVTAAGITLGMDELYKKKKKKKSKTKCVIM*

3) pIRES-mCerulean-GID1-Cdc42/N12-Cdc42/13C(Q61L)-GAI$_{1-92}$-mVenus-CAAX

> 61L is underlined.

MVSKGEELFTGVVPILVELDGDVNGHKFSVSGEGEGDATYGKLTLKFICTTGKLPVPWPTLVTT
LTWGVQCFARYPDHMKQHDFFKSAMPEGYVQERTIFFKDDGNYKTRAEVKFEGDTLVNRIELK
GIDFKEDGNILGHKLEYNAISDNVYITADKQKNGIKANFKIRHNIEDGSVQLADHYQQNTPIGDGP
VLLPDNHYLSTQSKLSKDPNEKRDHMVLLEFVTAAGITLGMDELYKGGGSSGGGMAASDEVNL
IESRTVVPLNTWVLISNFKVAYNILRRPDGTFNRHLAEYLDRKVTANANPVDGVFSFDVLIDRRIN
LLSRVYRPAYADQEQPPSILDLEKPVDGDIVPVILFFHGGSFAHSSANSAIYDTLCRRLVGLCKCV
VVSVNYRRAPENPYPCAYDDGWIALNWVNSRSWLKSKKDSKVHIFLAGDSSGGNIAHNVALRA
GESGIDVLGNILLNPMFGGNERTESEKSLDGKYFVTVRDRDWYWKAFLPEGEDREHPACNPF
SPRGKSLEGVSFPKSLVVVAGLDLIRDWQLAYAEGLKKAGQEVKLMHLEKATVGFYLLPNNNHF
HNVMDEISAFVNAECQISYASRGGGSSGGGELQTIKCVVVGDGA∗---***IRES Region***---
MGVGKTCLLISYTTNKFPSEYVPTVFDNYAVTVMIGGEPYTLGLFDTAGLEDYDRLRPLSYPQT
DVFLVCFSVVSPSSFENVKEKWVPEITHHCPKTPFLLVGTQIDLRDDPSTIEKLAKNKQKPITPET
AEKLARDLKAVKYVECSALTQRGLKNVFDEAILAALEPPETQPGDPNWELVYTARLQGGGSSG
GGQISYASRGMKRDHHHHHHQDKKTMMMNEEDDGNGMDELLAVLGYKVRSSEMADVAQKLE
QLEVMMSNVQEDDLSQLATETVHYNPAELYTWLDSMLTDLNGGGSSGGGVSKGEELFTGVVP
ILVELDGDVNGHKFSVSGEGEGDATYGKLTLKLICTTGKLPVPWPTLVTTLGYGLQCFARYPDH
MKQHDFFKSAMPEGYVQERTIFFKDDGNYKTRAEVKFEGDTLVNRIELKGIDFKEDGNILGHKL
EYNYNSHNVYITADKQKNGIKANFKIRHNIEDGGVQLADHYQQNTPIGDGPVLLPDNHYLSYQS
KLSKDPNEKRDHMVLLEFVTAAGITLGMDELYKKKKKKKSKTKCVIM∗

4) **pIRES-mCerulean-GID1-Cdc42/N12-Cdc42/13C(T17N)-GAI$_{1\text{-}92}$-mVenus-CAAX**

> <u>17N is underlined.</u>

MVSKGEELFTGVVPILVELDGDVNGHKFSVSGEGEGDATYGKLTLKFICTTGKLPVPWPTLVTT
LTWGVQCFARYPDHMKQHDFFKSAMPEGYVQERTIFFKDDGNYKTRAEVKFEGDTLVNRIELK
GIDFKEDGNILGHKLEYNAISDNVYITADKQKNGIKANFKIRHNIEDGSVQLADHYQQNTPIGDGP
VLLPDNHYLSTQSKLSKDPNEKRDHMVLLEFVTAAGITLGMDELYKGGGSSGGGMAASDEVNL
IESRTVVPLNTWVLISNFKVAYNILRRPDGTFNRHLAEYLDRKVTANANPVDGVFSFDVLIDRRIN
LLSRVYRPAYADQEQPPSILDLEKPVDGDIVPVILFFHGGSFAHSSANSAIYDTLCRRLVGLCKCV
VVSVNYRRAPENPYPCAYDDGWIALNWVNSRSWLKSKKDSKVHIFLAGDSSGGNIAHNVALRA
GESGIDVLGNILLNPMFGGNERTESEKSLDGKYFVTVRDRDWYWKAFLPEGEDREHPACNPF
SPRGKSLEGVSFPKSLVVVAGLDLIRDWQLAYAEGLKKAGQEVKLMHLEKATVGFYLLPNNNHF
HNVMDEISAFVNAECQISYASRGGGSSGGGELQTIKCVVVGDGA*---***IRES Region***---
MGVGK**<u>N</u>**CLLISYTTNKFPSEYVPTVFDNYAVTVMIGGEPYTLGLFDTAGQEDYDRLRPLSYPQT
DVFLVCFSVVSPSSFENVKEKWVPEITHHCPKTPFLLVGTQIDLRDDPSTIEKLAKNKQKPITPET
AEKLARDLKAVKYVECSALTQRGLKNVFDEAILAALEPPETQPGDPNWELVYTARLQGGGSSG
GGQISYASRGMKRDHHHHHHQDKKTMMMNEEDDGNGMDELLAVLGYKVRSSEMADVAQKLE
QLEVMMSNVQEDDLSQLATETVHYNPAELYTWLDSMLTDLNGGGSSGGGVSKGEELFTGVVP
ILVELDGDVNGHKFSVSGEGEGDATYGKLTLKLICTTGKLPVPWPTLVTTLGYGLQCFARYPDH
MKQHDFFKSAMPEGYVQERTIFFKDDGNYKTRAEVKFEGDTLVNRIELKGIDFKEDGNILGHKL
EYNYNSHNVYITADKQKNGIKANFKIRHNIEDGGVQLADHYQQNTPIGDGPVLLPDNHYLSYQS
KLSKDPNEKRDHMVLLEFVTAAGITLGMDELYKKKKKKKSKTKCVIM*

5) **pIRES-mCerulean-ABI-RhoA/N12-RhoA/13C(Q63L)-PYL-mVenus-CAAX**

> 63L is underlined.

MVSKGEELFTGVVPILVELDGDVNGHKFSVSGEGEGDATYGKLTLKFICTTGKLPVPWPTLVTT
LTWGVQCFARYPDHMKQHDFFKSAMPEGYVQERTIFFKDDGNYKTRAEVKFEGDTLVNRIELK
GIDFKEDGNILGHKLEYNAISDNVYITADKQKNGIKANFKIRHNIEDGSVQLADHYQQNTPIGDGP
VLLPDNHYLSTQSKLSKDPNEKRDHMVLLEFVTAAGITLGMDELYKGGGSSGGGVPLYGFTSIC
GRRPEMEAAVSTIPRFLQSSSGSMLDGRFDPQSAAHFFGVYDGHGGSQVANYCRERMHLALA
EEIAKEKPMLCDGDTWLEKWKKALFNSFLRVDSEIESVAPETVGSTSVVAVVFPSHIFVANCGD
SRAVLCRGKTALPLSVDHKPDREDEAARIEAAGGKVIQWNGARVFGVLAMSRSIGDRYLKPSII
PDPEVTAVKRVKEDDCLILASDGVWDVMTDEEACEMARKRILLWHKKNAVAGDASLLADERRK
EGKDPAAMSAAEYLSKLAIQRGSKDNISVVVVDLKQISYASRGGGSSGGGELAAIRKKLVIVGD
GA*---***IRES Region***---
MCGKTCLLIVFSKDQFPEVYVPTVFENYVADIEVDGKQVELALWDTAG**L**EDYDRLRPLSYPDTD
VILMCFSIDSPDSLENIPEKWTPEVKHFCPNVPIILVGNKKDLRNDEHTRRELAKMKQEPVKPEE
GRDMANRIGAFGYMECSAKTKDGVREVFEMATRAALQAGDPNWELVYTARLQGGGSSGGGQ
ISYASRGTQDEFTQLSQSIAEFHTYQLGNGRCSSLLAQRIHAPPETVWSVVRRFDRPQIYKHFIK
SCNVSEDFEMRVGCTRDVNVISGLPANTSRERLDLLDDDRRVTGFSITGGEHRLRNYKSVTTV
HRFEKEEEEERIWTVVLESYVVDVPEGNSEEDTRLFADTVIRLNLQKLASITEAMNGGGSSGGG
VSKGEELFTGVVPILVELDGDVNGHKFSVSGEGEGDATYGKLTLKLICTTGKLPVPWPTLVTTLG
YGLQCFARYPDHMKQHDFFKSAMPEGYVQERTIFFKDDGNYKTRAEVKFEGDTLVNRIELKGI
DFKEDGNILGHKLEYNYNSHNVYITADKQKNGIKANFKIRHNIEDGGVQLADHYQQNTPIGDGP
VLLPDNHYLSYQSKLSKDPNEKRDHMVLLEFVTAAGITLGMDELYKKKKKKKSKTKCVIM*

6) **pIRES-mCerulean-ABI-RhoA/N12-RhoA/13C(T19N)-PYL-mVenus-CAAX**

> 19N is underlined.

MVSKGEELFTGVVPILVELDGDVNGHKFSVSGEGEGDATYGKLTLKFICTTGKLPVPWPTLVTT
LTWGVQCFARYPDHMKQHDFFKSAMPEGYVQERTIFFKDDGNYKTRAEVKFEGDTLVNRIELK
GIDFKEDGNILGHKLEYNAISDNVYITADKQKNGIKANFKIRHNIEDGSVQLADHYQQNTPIGDGP
VLLPDNHYLSTQSKLSKDPNEKRDHMVLLEFVTAAGITLGMDELYKGGGSSGGGVPLYGFTSIC
GRRPEMEAAVSTIPRFLQSSSGSMLDGRFDPQSAAHFFGVYDGHGGSQVANYCRERMHLALA
EEIAKEKPMLCDGDTWLEKWKKALFNSFLRVDSEIESVAPETVGSTSVVAVVFPSHIFVANCGD
SRAVLCRGKTALPLSVDHKPDREDEAARIEAAGGKVIQWNGARVFGVLAMSRSIGDRYLKPSII
PDPEVTAVKRVKEDDCLILASDGVWDVMTDEEACEMARKRILLWHKKNAVAGDASLLADERRK
EGKDPAAMSAAEYLSKLAIQRGSKDNISVVVVDLKQISYASRGGGSSGGGELAAIRKKLVIVGD
GA*---***IRES Region***---
MCGK**N**CLLIVFSKDQFPEVYVPTVFENYVADIEVDGKQVELALWDTAGQEDYDRLRPLSYPDT
DVILMCFSIDSPDSLENIPEKWTPEVKHFCPNVPIILVGNKKDLRNDEHTRRELAKMKQEPVKPE
EGRDMANRIGAFGYMECSAKTKDGVREVFEMATRAALQAGDPNWELVYTARLQGGGSSGGG
QISYASRGTQDEFTQLSQSIAEFHTYQLGNGRCSSLLAQRIHAPPETVWSVVRRFDRPQIYKHFI
KSCNVSEDFEMRVGCTRDVNVISGLPANTSRERLDLLDDDRRVTGFSITGGEHRLRNYKSVTT
VHRFEKEEEEEERIWTVVLESYVVDVPEGNSEEDTRLFADTVIRLNLQKLASITEAMNGGGSSGG
GVSKGEELFTGVVPILVELDGDVNGHKFSVSGEGEGDATYGKLTLKLICTTGKLPVPWPTLVTTL
GYGLQCFARYPDHMKQHDFFKSAMPEGYVQERTIFFKDDGNYKTRAEVKFEGDTLVNRIELKG
IDFKEDGNILGHKLEYNYNSHNVYITADKQKNGIKANFKIRHNIEDGGVQLADHYQQNTPIGDGP
VLLPDNHYLSYQSKLSKDPNEKRDHMVLLEFVTAAGITLGMDELYKKKKKKKSKTKCVIM*

7) **pMAL-MBP-GID1-Cdc42/N12**

MKIEEGKLVIWINGDKGYNGLAEVGKKFEKDTGIKVTVEHPDKLEEKFPQVAATGDGPDIIFWAH
DRFGGYAQSGLLAEITPDKAFQDKLYPFTWDAVRYNGKLIAYPIAVEALSLIYNKDLLPNPPKTW
EEIPALDKELKAKGKSALMFNLQEPYFTWPLIAADGGYAFKYENGKYDIKDVGVDNAGAKAGLT
FLVDLIKNKHMNADTDYSIAEAAFNKGETAMTINGPWAWSNIDTSKVNYGVTVLPTFKGQPSKP
FVGVLSAGINAASPNKELAKEFLENYLLTDEGLEAVNKDKPLGAVALKSYEEELVKDPRIAATME
NAQKGEIMPNIPQMSAFWYAVRTAVINAASGRQTVDEALKDAQTNSSSNNNNNNNNNNLGIEG
RISHMAASDEVNLIESRTVVPLNTWVLISNFKVAYNILRRPDGTFNRHLAEYLDRKVTANANPVD
GVFSFDVLIDRRINLLSRVYRPAYADQEQPPSILDLEKPVDGDIVPVILFFHGGSFAHSSANSAIY
DTLCRRLVGLCKCVVVSVNYRRAPENPYPCAYDDGWIALNWVNSRSWLKSKKDSKVHIFLAGD
SSGGNIAHNVALRAGESGIDVLGNILLNPMFGGNERTESEKSLDGKYFVTVRDRDWYWKAFLP
EGEDREHPACNPFSPRGKSLEGVSFPKSLVVVAGLDLIRDWQLAYAEGLKKAGQEVKLMHLEK
ATVGFYLLPNNNHFHNVMDEISAFVNAECQISYASRGGGSSGGGELQTIKCVVVGDGA*

8) **pET21b-Cdc42/13C-GAI$_{1-92}$-MBP**

MGVGKTCLLISYTTNKFPSEYVPTVFDNYAVTVMIGGEPYTLGLFDTAGLEDYDRLRPL
SYPQTDVFLVCFSVVSPSSFENVKEKWVPEITHHCPKTPFLLVGTQIDLRDDPSTIEKLA
KNKQKPITPETAEKLARDLKAVKYVECSALTQRGLKNVFDEAILAALEPPETQPGDPNW
ELVYTARLQGGGSSGGGQISYASRGMKRDHHHHHHQDKKTMMMNEEDDGNGMDELLAV
LGYKVRSSEMADVAQKLEQLEVMMSNVQEDDLSQLATETVHYNPAELYTWLDSMLTDLNKLE
NLYFQGEEGKLVIWINGDKGYNGLAEVGKKFEKDTGIKVTVEHPDKLEEKFPQVAATGD
GPDIIFWAHDRFGGYAQSGLLAEITPDKAFQDKLYPFTWDAVRYNGKLIAYPIAVEALSLI
YNKDLLPNPPKTWEEIPALDKELKAKGKSALMFNLQEPYFTWPLIAADGGYAFKYENG
KYDIKDVGVDNAGAKAGLTFLVDLIKNKHMNADTDYSIAEAAFNKGETAMTINGPWAWS
NIDTSKVNYGVTVLPTFKGQPSKPFVGVLSAGINAASPNKELAKEFLENYLLTDEGLEAV
NKDKPLGAVALKSYEEELAKDPRIAATMENAQKGEIMPNIPQMSAFWYAVRTAVINAAS
GRQTVDEALKDAQTNSSS*

Reference

(1) Jacquemet, G.; Hamidi, H.; Ivaska, J. Filopodia quantification using filoquant. *Methods Mol. Biol.* **2019**, 2040,359-373.
(2) Gibson, D. G.; Young, L.; Chuang, R.-Y.; Venter, J. C.; Hutchison, C. A.; Smith, H. O. Enzymatic assembly of DNA molecules up to several hundred kilobases. *Nat. Methods* **2009**, 6, 343-345.
(3) He, Y.; Faulkner, B. M.; Roberti, M. A.; Bassford, D. K.; Stains, C. I. Standardized parts for activation of small GTPase signaling in living Cells. *Angew. Chem. Int. Ed.* **2024***,* 63, e202403499.
(4) Liang, F.-S.; Ho, W. Q.; Crabtree, G. R. Engineering the ABA plant stress pathway for regulation of induced proximity. *Sci. Signal.* **2011**, *4*, rs2-rs2.
(5) Miyamoto, T.; DeRose, R.; Suarez, A.; Ueno, T.; Chen, M.; Sun, T.-p.; Wolfgang, M. J.; Mukherjee, C.; Meyers, D. J.; Inoue, T. Rapid and orthogonal logic gating with a gibberellin-induced dimerization system. *Nat. Chem. Biol.* **2012**, *8*, 465-470.
(6) Jacquemet, G.; Paatero, I.; Carisey, A. F.; Padzik, A.; Orange, J. S.; Hamidi, H.; Ivaska, J. FiloQuant reveals increased filopodia density during breast cancer progression. *J. Cell Biol.* **2017**, 216, 3387-3403.
